# Nitrogen Doping of Confined Carbyne

**DOI:** 10.1021/acs.jpclett.5c01063

**Published:** 2025-05-12

**Authors:** Clara Freytag, Christin Schuster, Weili Cui, Nikos Tagmatarchis, Rubén Cantón-Vitoria, Lei Shi, Emil Parth, Kazuhiro Yanagi, Paola Ayala, Thomas Pichler

**Affiliations:** † 27258 University of Vienna, Faculty of Physics, Boltzmanngasse 5, 1090 Vienna, Austria; ‡ State Key Laboratory of Optoelectronic Materials and Technologies, Guangdong Basic Research Center of Excellence for Functional Molecular Engineering, Nanotechnology Research Center, School of Materials Science and Engineering, 26469 Sun Yat-sen University, Guangzhou 510275, China; § Theoretical and Physical Chemistry Institute, 54574 National Hellenic Research Foundation, 48, Vasileos Constantinou Avenue, 11635 Athens, Greece; ∥ Department of Physics, Tokyo Metropolitan University, 192-0397 Tokyo, Japan

## Abstract

Low-dimensional carbon allotropes belong to the most revolutionary materials of the most recent decades. Confined carbyne, a linear chain of sp^1^-hybridized carbon encapsulated inside a small-diameter carbon nanotube host, is one extraordinary nanoengineering example. Inspired by these hybrid structures, we demonstrate the feasibility to synthesize nitrogen-doped confined carbyne by using azafullerenes (C_59_N) encapsulated in nanotubes (“peapods”) as precursors for the growth of confined carbyne. Resonance Raman spectroscopy as a site selective local probe has served to identify the changes in the spectra of nitrogen-doped versus pristine carbon peapods and confined carbyne. We are able to disentangle frequency changes due to charge transfer from changes due to the difference in mass for both the nanotube and the carbyne, where different effects dominate. This study demonstrates a suitable pathway to achieve controlled doping of carbyne chains via the use of specifically doped precursors.

## Graphical abstract

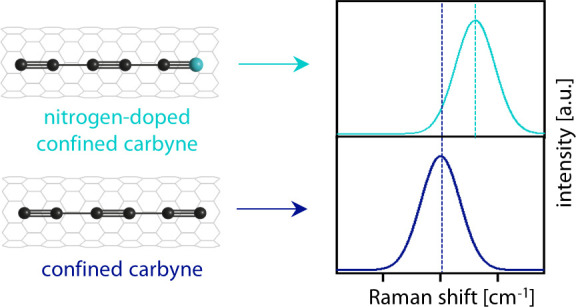

Carbyne is a material consisting of an infinitely long linear chain of sp^1^-hybridized carbon atoms with extraordinary predicted electronic, optical and mechanical properties.^1–8^ In general, linear chains of carbon can have two different bonding geometries, exhibiting either continuous double bonds (cumulenic structure) or alternating single and triple bonds (polyynic structure), with the latter being more energetically favorable due to Peierl’s distortion.^9^ A linear carbon chain is only considered infinite when the properties of the chain become independent of the chain length; otherwise, they are referred to as polyynes or cumulenes, depending on the bonding situation.^10^ Freestanding carbyne is unstable due to exothermic collapse of the chain or cross-linking with other chains.^11^ This explains the breakthrough in stabilizing carbyne chains by synthesizing them directly inside double-walled carbon nanotubes (DWCNTs).^12^ The interaction of the chain with the surrounding CNT stabilizes it and prevents cross-linking or collapse of the chain onto itself. Hereafter, we will refer to the hybrid material of carbyne inside carbon nanotube hosts as confined carbyne (CC). The main synthesis method of CC involves high-temperature, high-vacuum annealing of CNTs.^13–15^ However, low-temperature annealing^16–18^ and photothermal synthesis^19^ have also been successfully applied. Investigations into the growth mechanisms have shown that CC grows from carbonaceous precursors inside the nanotube hosts that exist from the CNT synthesis process and can be increased by filling the nanotubes with, e.g., small organic molecules,^20^ short-chain polyynes,^21^ or fullerenes.^15^ Through isotopic labeling of C_60_ filled into ultra-clean single-walled carbon nanotubes (SWCNTs), it was shown that the inner tube and the chain grow from the fullerenes, and there is also an exchange of carbon atoms between the inner and outer tube and the chain.^22^ Furthermore, the possibility to encapsulate azafulleneres into SWCNTs has been demonstrated previously^23^ and opens more possibilities for exploiting the incorporation of nitrogen as a dopant into nanotubes and confined carbyne.

The primary method to study CC is Raman spectroscopy, since carbyne is an exceptional Raman scatterer.^24^ Additionally, shifts in the Raman frequency can be observed for doped CNTs,^25^ making it the ideal technique to study doped CC. There are two concomitant effects which contribute to a shift in the Raman frequency of CNTs. First, the change in mass of the dopant influences the frequency according to eq 1, where ν is the frequency of the doped nanotube, ν_0_ the frequency of the undoped nanotube, *m*_0_ and *m*_*x*_ are the atomic masses of carbon and the dopant, and *c* is the relative concentration of the dopant.^26^

1 $$\frac{\nu_{0}-\nu }{\nu_{0}}=1-\sqrt{\frac{m_{0}}{m_{0}+\left(m_{x}-m_{0}\right)c}}$$

Additionally, changes in the electronic structure due to charge transfer lead to shifts in the Raman frequency of the G band in graphene to higher wavenumbers, regardless of p-type or n-type doping. Both the increase and decrease of the surface electron concentration leads to an upshift of the G band frequency, which has been demonstrated theoretically and experimentally.^27,28^ This has been attributed to nonadiabitic effects and a breakdown of the Born–Oppenheimer approximation. Upshifts in the G mode of graphite have also been observed for graphite intercalation compounds for moderate levels of intercalant.^29^ Further, for intercalated graphite, downshifts of the frequency of the G mode and 2D mode were attributed to strain in the lattice. For the 2D mode, only the downshift effect from the strain is observed, as this mode originates, as opposed to the G band, from the *K* point rather than the Γ point, where the charge transfer affects the band structure. Downshifts in the 2D mode were also observed for nitrogen-doped graphene in moderate concentrations.^30^ It is hard to disentangle these effects quantitatively, especially as they lead to a shift in the Raman frequency in different directions. Despite the difficulties in quantifying the doping-related effects on the Raman response of these hybrid materials, it is clearly feasible to pinpoint the predominant effects related to the net change of the Raman shift.

For the experiments, semiconducting arc-discharge CNTs (diameter, 1.36 ± 0.08 nm) were used as SWCNT hosts. These host tubes were filled with C_60_ and C_59_N fullerenes, respectively, in vacuum to create the doped and undoped peapods. The peapods were then annealed at high temperature in high vacuum to create DWCNTs and, in a second high temperature, high vacuum annealing step, CC@DWCNTs. Raman spectra were taken after the DWCNTs were made and after the final annealing step. The synthesis pathway is schematically shown in Figure 1; the exact experimental conditions for each step are described in the Supporting Information.

**Figure 1:**
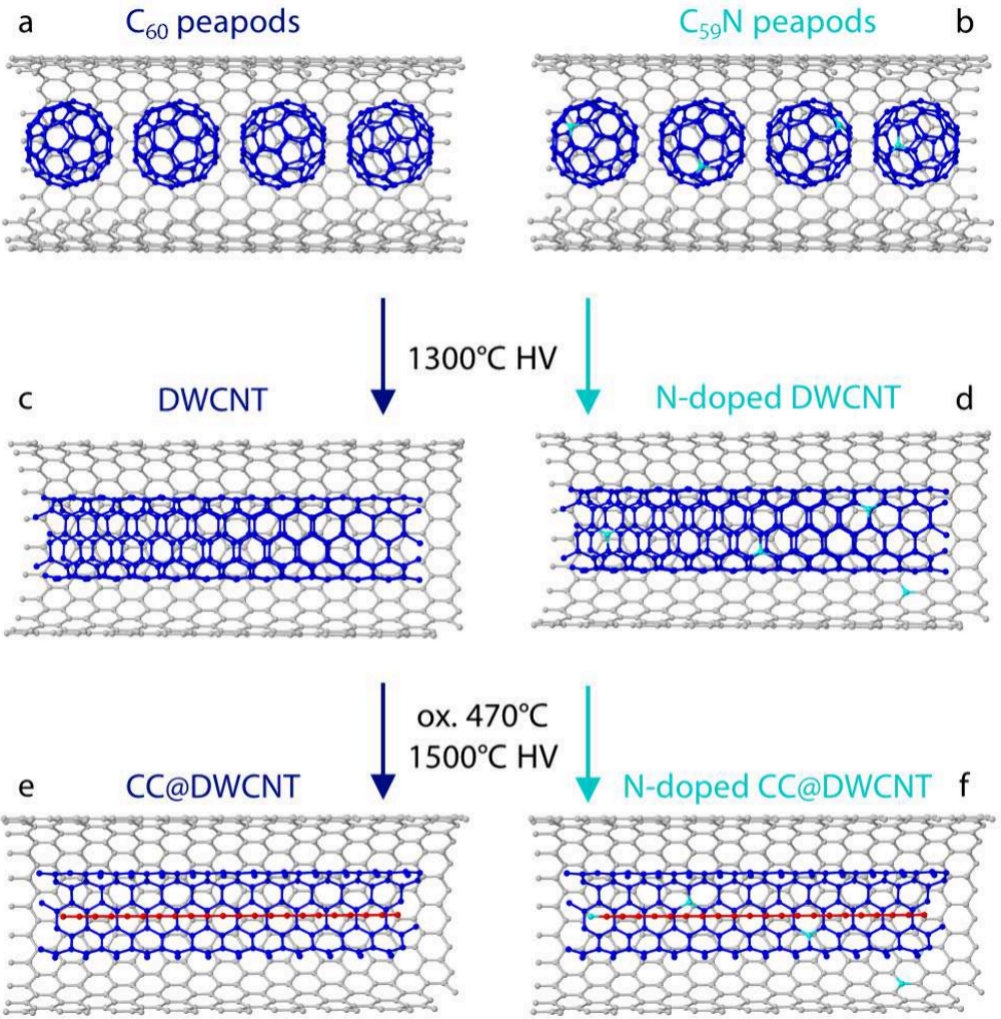
Synthesis of undoped (left) and nitrogen-doped (right) confined carbyne: (a) C_60_ inside a SWCNT; (b) C_59_N inside a SWCNT; (c) undoped DWCNT; (d) nitrogen-doped DWCNT; (e) undoped CC@DWCNT; (f) nitrogen-doped CC@DWCNT.

The presence of the respective fullerenes inside the SWCNTs after filling was confirmed using Raman spectroscopy, where the A_g_(2) modes of C_60_ and C_59_N at ∼1465 and ∼1455 cm^–1^, respectively, were observed (Figure 2a). The spectrum of the doped fullerene peapods shows a significant downshift in the Raman frequency, confirming the presence of the doped species inside the SWCNTs.

**Figure 2:**
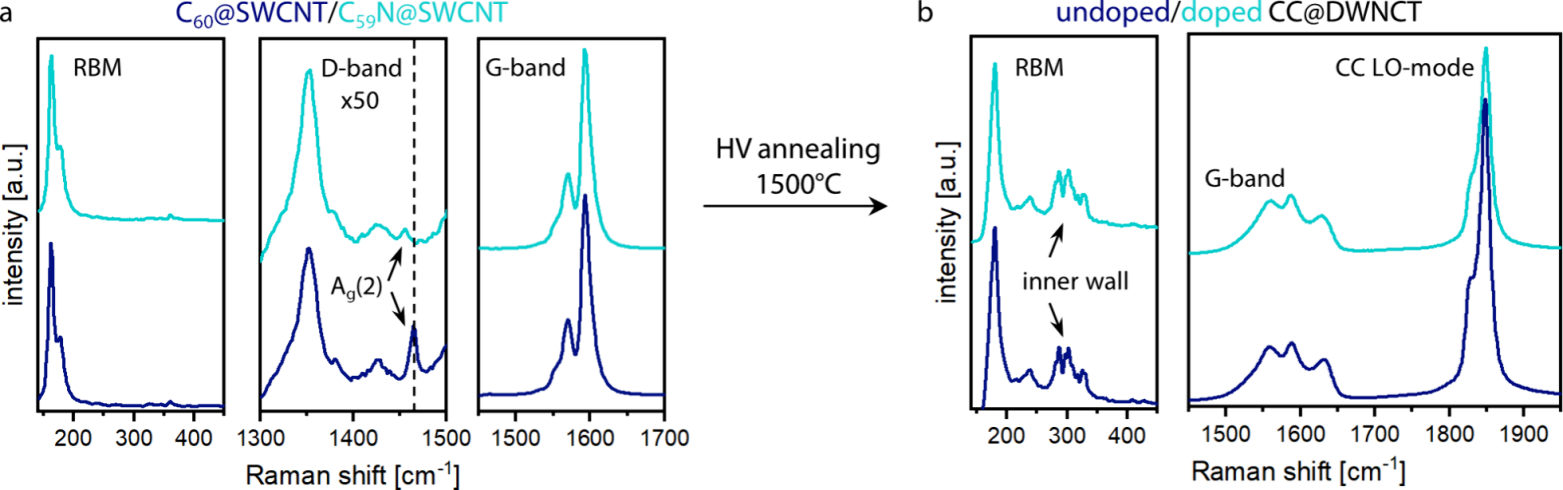
(a) Raman spectra of C_60_ (dark blue) and C_59_N (teal) peapods, measured with a 488 nm laser (in resonance with the fullerene A_g_(2) mode). The shift of the A_g_(2) mode is highlighted. (b) Raman spectra of doped/undoped CC@DWCNTs, measured with a 568 nm laser (in resonance with the CC).

As an intermediate step, DWCNTs were grown from the C_60_ and C_59_N peapods and Raman spectra were recorded (Figure 3). The Raman spectra of the G mode at ∼1595 cm^–1^ of the doped and undoped samples were compared. A small downshift can be observed for the sample grown from the doped fullerenes. In order to make an estimation of the shifts for the DWCNTs grown from C_60_ and C_59_N fullerenes, a line-shape analysis was performed. It was observed that for the nitrogen-doped system, for the G mode (Figure 3a,c), almost all peaks are shifted toward lower wavenumbers by 1–3 cm^–1^, as it would be expected for doping with an atom with a higher mass. The increase in surface electron concentration from the nitrogen concentration in the fullerenes would not lead to strong shifts in the Raman frequency according to the calculation in Pisana et al.,^27^ which means that, in this case, the effect of the mass is dominant. For the 2D peak of the DWCNTs, even larger downshifts of 6–10 cm^–1^ were observed for the doped DWCNTs (Figure 3b,d). The shift in the 2D line is expected to result only from the mass effect and strain.^29^ Similar shifts in the 2D mode have been previously observed for DWCNTs grown from C_59_N peapods.^31^

**Figure 3:**
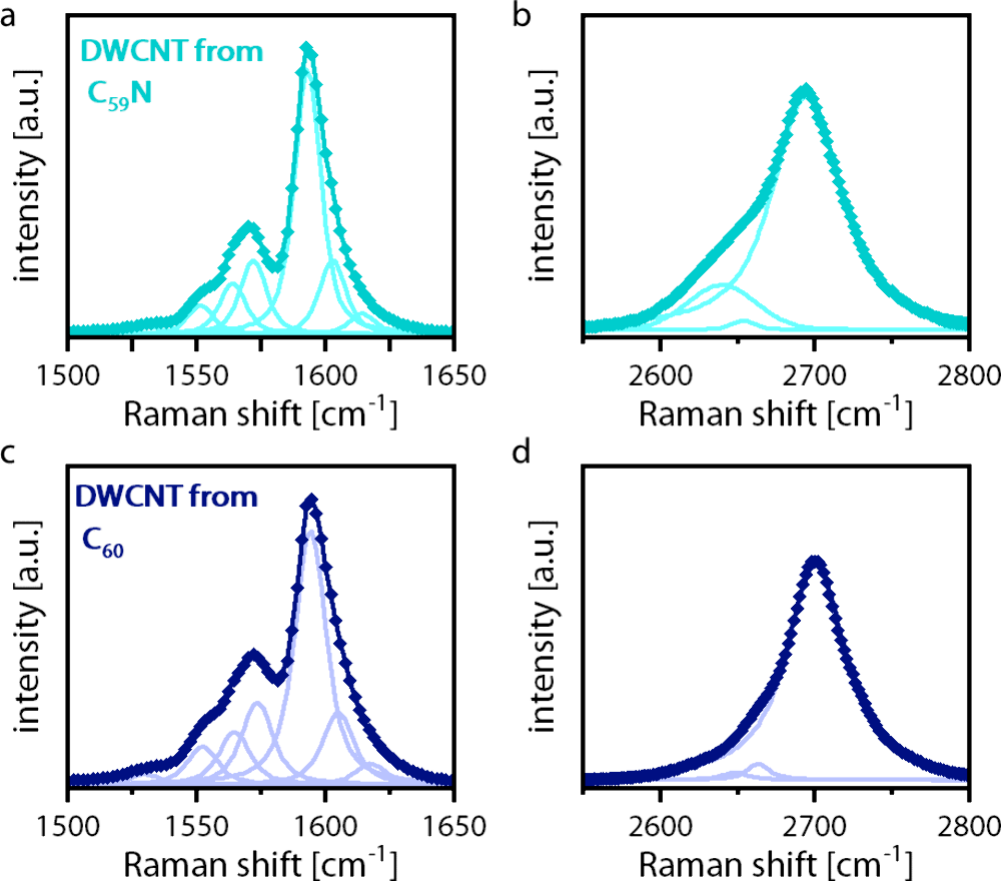
Raman spectra of DWCNTs grown from C_60_ (dark blue) and C_59_N (teal) peapods: (a) line-shape analysis of the G mode of doped DWCNTs; (b) line-shape analysis of the 2D mode of doped DWCNTs; (c) line-shape analysis of the G mode of undoped DWCNTs; (d) line-shape analysis of the 2D mode of undoped DWCNTs.

After the synthesis of CC, the LO mode assigned to the CC at ∼1850 cm^–1^ is observed in the Raman spectra for both the doped and the undoped sample (Figure 2b) with similar intensity. Additionally, the changes in the G mode, which have recently been described by anharmonic phonon–phonon interactions of the CC with the hosting DWCNT,^32^ confirm the presence of CC.

The CC mode can be fitted with multiple components due to the different Raman frequencies of CC present in the sample, depending on the host nanotube geometry.^33^ According to the estimations made in the work of Heeg et al., the predominant inner tube chiralities and diameters in these samples were assigned to (6,5/0.75 nm), (7,4/0.76 nm), (8,3/0.78 nm), and (9,2/0.80 nm). Since the same single-walled hosts were used for both samples, the same inner tube chiralities can be expected to grow and the peak positions of the components can be compared in order to analyze shifts due to doping. The line-shape analysis for the CC modes of the undoped and doped sample is shown in Figure 4a. The Raman shifts of the four components relative to the undoped sample are plotted in Figure 4b. Multiple spots were measured on each sample. The components of the undoped sample are all at very similar Raman shifts, with only a small deviation (4 spots measured). The shifts in the frequency of the CC peaks of the doped sample are shifted to higher wavenumbers by an average of around 3 cm^–1^, and they show a higher variance (10 spots measured). The higher variance is to be expected as the nitrogen is not necessarily homogeneously distributed in the sample.

**Figure 4:**
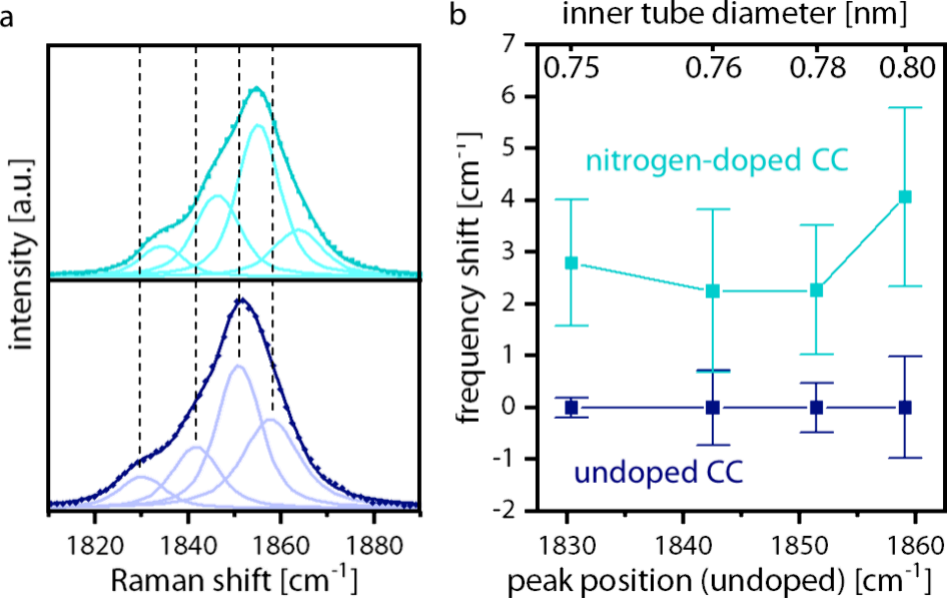
Analysis of CC grown from nitrogen-doped fullerenes (teal) and undoped fullerenes (dark blue): (a) line-shape analysis of the CC mode of the undoped sample compared to the doped sample; (b) average frequency shifts of components of the doped sample (10 different spots measured) compared to the undoped sample (4 different spots measured).

To interpret the shifts observed for the Raman frequency of the CC, we must consider the two effects of mass change and charge transfer mentioned above that contribute to shifts in the Raman frequency. On one hand, the frequency of a vibration is inversely proportional to the masses involved, leading to an increase in the frequency for lower masses and a decrease for higher masses. On the other hand, electronic doping leads to upward shifts in the Raman frequency, regardless of whether the structure is depleted of electrons (p-type doping) or electrons are added (n-type doping). In the case of graphene according to the estimations made in Pisana et al.^27^ and Lazzeri et al.,^28^ the small amount of doping achieved in our case would not lead to significant shifts in the Raman frequency of the G mode. We can, however, assume that due to the confinement to one dimension compared to graphene, the effect of additional charges in the system is stronger. Disentangling the two effects and evaluating the dopant concentration are not trivial. In our case, the effect from charge transfer dominates the effect of the mass on the Raman frequency, since only shifts to higher frequencies were observed.

The nitrogen bonding configuration within the chain can have multiple possibilities. Assuming that nitrogen would form three covalent bonds, it could be expected that it would terminate fragments of CC. The carbon atoms in the middle of the chain all have four covalent bonds (alternating single and triple bonds), which means that the chain end would be ideal for a three-valent atom, preventing dangling bonds at the end of the carbyne chain. Short-chain nitrogen-capped polyynes have been successfully isolated before by introducing nitrogen in the carrier gas during laser vaporization of graphite, showing that such a structure would be stable.^34^ Cyanopolyynes with up to 12 carbon atoms in the chain have also been isolated and studied in solution.^35^ Another theoretical possibility would be for the nitrogen to be coordinated in a pyridinic way, with two carbons bonded on either side, one with a double bond and one with a single bond. This would, however, disrupt the more stable polyynic character (alternating single and triple bonds) of the chain and impose a cumulenic structure (continuous double bonds) on one side. Additionally, due to the lone electron pair of the nitrogen, the bonding geometry would not be linear but would introduce a kink into the chain. Due to the strong spatial confinement in the small-diameter host tubes, this is unlikely.

In conclusion, nitrogen atoms were successfully introduced into the carbyne/nanotube system by means of filling the carbon nanotube host tubes with a nitrogen-doped precursor. The presence of nitrogen atoms in the chain was indirectly confirmed by shifts in the Raman frequencies of the peaks related to the carbyne chain. Different effects dominate the shifts in the Raman frequencies of the chain and the surrounding host tubes. To the best of our knowledge, this is the first report of the introduction of heteroatoms into confined carbyne structures.

## Supplementary Material



## Notes

Experimental details; tables containing peak positions from the line-shape analysis of the G mode and 2D mode of doped and undoped DWCNTs (PDF)
