# Supplementary material for: Nitrogen Doping of Confined Carbyne
Source: J Phys Chem Lett. 2025 May 12;16(20):4990–4. doi: 10.1021/acs.jpclett.5c01063 (PMC12105017; doi:10.1021/acs.jpclett.5c01063)
Supplement: Supplementary file 1 [file jz5c01063_si_001.pdf]

Nitrogen doping of confined carbyne - Supporting information

Clara Freytag,*,† Christin Schuster,† Weili Cui,‡ Nikos Tagmatarchis,¶ Rubén Cantón-Vitoria,¶ Lei Shi,‡ Emil Parth,† Kazuhiro Yanagi,§ Paola Ayala,† and Thomas Pichler*,†

†*University of Vienna, Faculty of Physics, Boltzmanngasse 5, 1090 Vienna, Austria*

‡*State Key Laboratory of Optoelectronic Materials and Technologies, Guangdong Basic Research Center of Excellence for Functional Molecular Engineering, Nanotechnology Research Center, School of Materials Science and Engineering, Sun Yat-sen University, Guangzhou 510275, China*

¶*National Hellenic Research Foundation, Theoretical and Physical Chemistry Institute, 48, Vasileos Constantinou Ave., 11635 Athens, Greece*

§*Department of Physics, Tokyo Metropolitan University, 192-0397 Tokyo, Japan*

E-mail: clara.freytag@univie.ac.at; thomas.pichler@univie.ac.at

Experimental details

For the experiments, semi-conducting arc-discharge CNTs (diameter 1.36±0.08 nm) were used. The diameter distribution was determined by analyzing the radial breathing modes (discussed in Parth *et al.*).

The doped and undoped CC@DWCNT samples shown in Figure 2 (main text) were synthesized using the following procedure. The synthesis began with the oxidation of semi-conducting arc-discharge nanotubes in air at 450 °C for 30 minutes to open their caps.

Subsequently, the C_{60} or the $C_{59}N$ powder was combined with the cap-opened nanotubes and sealed under vacuum (10^{-7} mbar) within a glass tube. The sealed glass tube was then heated to 500 °C for 72 hours to obtain the C_{60} and the $C_{59}N$ peapods. To remove excess, non-encapsulated fullerenes from the outside of the CNTs, they are shortly heated to 600°C in a vacuum furnace. Following the vapor filling, the C_{60} and the $C_{59}N$ peapods were subjected to high vacuum annealing at 1300 °C to grow inner tubes. This was followed by an additional heating step at 1600 °C to facilitate the formation of confined carbyne inside the inner tubes.

For the analysis performed in Figures 3 and 4 (main text), the samples were synthesized using the following procedure. Before filling with fullerenes, the nanotubes were oxidized in air at 450°C for 30 minutes to open the end caps. The C_{60} and the $C_{59}N$ fullerenes were each sublimated for 30 minutes at 550°C inside a glass ampoule. For the doped fullerenes, the dimer $(C_{59}N)_2$ was used in the process and this material is sublimed in monomeric form, as a $C_{59}N^{\cdot}$ radical, while the C_{60} is already a monomer in the starting material. The CNT samples were then sealed into glass ampoules under high vacuum with the doped fullerenes or undoped fullerenes. In order to fill the fullerenes into the nanotubes, the ampoules were placed in a two-zone furnace for 72 hours. The temperature at the fullerene side was 560°C for the $C_{59}N$ and 600°C for the C_{60} fullerenes and 370°C on the side of the carbon nanotubes for both ampoules. The next step was annealing the nanotube samples at 1300°C in high vacuum in order to grow double-walled carbon nanotubes (DWCNT). Both samples were annealed at 1500°C in high vacuum for 1 hour, oxidized at 470°C for 1 hour at 600 mbar of air and then annealed again at 1500°C in high vacuum for 1 hour.

The Raman measurements of all samples were made with a Horiba LabRAM HR spectrometer combined with an Ar^+/Kr^+ laser (Coherent Innova 70C). As detector, a liquid nitrogen cooled CCD chip was used. The spectra were calibrated using a Neon lamp.

Analysis of doped and undoped DWCNTs

Table S1: Raman frequencies of components of the G-mode fitted for double-walled carbon nanotubes made from undoped and doped precursors. The peaks marked with * are attributed to the G$^-$ peak of the outer nanotube, the peaks marked with † are attributed to the G$^+$ peak of the outer nanotube.

C_{60} [cm^{-1}]	$C_{59}N$ [cm^{-1}]	Difference [cm^{-1}]
1529.80	1530.76	+0.96
1552.19	1551.48	-0.71
1564.59	1563.88	-0.71
1573.46*	1571.88*	-1.58
1594.04†	1592.95†	-1.09
1604.85	1602.66	-2.19
1617.33	1614.19	-3.17

Table S2: Raman frequencies of components of the 2D-mode fitted for double-walled carbon nanotubes made from undoped and doped precursors.

C_{60} [cm^{-1}]	$C_{59}N$ [cm^{-1}]	Difference [cm^{-1}]
2647.28	2639.62	-7.66
2663.22	2653.59	-9.63
2700.28	2694.39	-5.89
